# Assessment of *MYC* and *TERT* copy number variations in lung cancer using digital PCR

**DOI:** 10.1186/s13104-023-06566-x

**Published:** 2023-10-19

**Authors:** Alexander Brik, Katharina Wichert, Daniel G. Weber, Katja Szafranski, Peter Rozynek, Swetlana Meier, Yon-Dschun Ko, Reinhard Büttner, Klaus Gerwert, Thomas Behrens, Thomas Brüning, Georg Johnen

**Affiliations:** 1 Institute for Prevention and Occupational Medicine of the German Social Accident Insurance – Institute of the Ruhr University Bochum (IPA), Bochum, Germany; 2 Department of Internal Medicine, Johanniter-Kliniken Bonn GmbH, Bonn, Germany; 3 Institute of Pathology, Medical Faculty and Center for Molecular Medicine (CMMC), University of Cologne, Cologne, Germany; 4 Center for Protein Diagnostics (PRODI), Department of Biophysics, Ruhr University Bochum, Bochum, Germany

**Keywords:** Marker combination, Copy number variations, Digital polymerase chain reaction, Diagnosis, Non-small cell lung cancer, Frozen tissue

## Abstract

**Objective:**

Lung cancer is the second most frequent cancer type and the most common cause of cancer-related deaths worldwide. Alteration of gene copy numbers are associated with lung cancer and the determination of copy number variations (CNV) is appropriate for the discrimination between tumor and non-tumor tissue in lung cancer. As telomerase reverse transcriptase (*TERT*) and v-myc avian myelocytomatosis viral oncogene homolog (*MYC*) play a role in lung cancer the aims of this study were the verification of our recent results analyzing *MYC* CNV in tumor and non-tumor tissue of lung cancer patients using an independent study group and the assessment of *TERT* CNV as an additional marker.

**Results:**

*TERT* and *MYC* status was analyzed using digital PCR (dPCR) in tumor and adjacent non-tumor tissue samples of 114 lung cancer patients. The difference between tumor and non-tumor samples were statistically significant (p < 0.0001) for *TERT* and *MYC*. Using a predefined specificity of 99% a sensitivity of 41% and 51% was observed for *TERT* and *MYC*, respectively. For the combination of *TERT* and *MYC* the overall sensitivity increased to 60% at 99% specificity. We demonstrated that a combination of markers increases the performance in comparison to individual markers. Additionally, the determination of CNV using dPCR might be an appropriate tool in precision medicine.

**Supplementary Information:**

The online version contains supplementary material available at 10.1186/s13104-023-06566-x.

## Introduction

Lung cancer constitutes a major health burden. Worldwide, 2,206,771 new cases and 1,796,144 deaths were assigned to lung cancer in 2020, representing the second most frequent cancer type (11.4%) and the most common cause of cancer-related deaths (18.0%) [1].

The major histological lung cancer group (~ 85%) is non-small cell lung cancer (NSCLC). NSCLC shows a 5-year survival of 10% for patients at advanced stages in comparison to 68% for patients at early stages [2, 3].

Many cancers exhibit chromosomal instability resulting in amplification/gain or loss/deletion of genomic DNA. Copy number variation (CNV) is characterized by a change of DNA sequence number in comparison to the normal (diploid) genome [4]. Lung cancer as well as several other cancers are associated with CNV [5, 6].

Recently, we have shown that the determination of v-myc avian myelocytomatosis viral oncogene homolog (*MYC*) CNV is an appropriate tool to discriminate between lung tumor and non-tumor tissues with a sensitivity of 43% and 99% specificity [7]. *MYC* is located on chromosome 8q24.21 and encodes a transcription factor playing a role in cell cycle, apoptosis, and cellular transformation. Notably, *MYC* is one of the most frequently amplified genes in lung cancer [8].

Similarly, telomerase reverse transcriptase (*TERT*) is frequent amplified in early-stage lung cancer [9–11]. Barthel et al. observed a *TERT* amplification in 13% and 14% of lung adenocarcinoma (LUAD) and lung squamous cell carcinoma (LSCC) cases, respectively [12]. *TERT* is located on chromosome 5p15.33 and is a major component of the telomerase complex. Up to 90% of all tumors are marked by telomerase activation [13, 14]*.* Cao et al. proposed that an increase of the *TERT* copy number is an important mechanism for upregulation of telomerase activity in human cancer [15]. *TERT* is a key player supporting immortality of cancer cells and the amplification of *TERT* with other chromosomal aberrations plays a role in tumor development and progression [16, 17].

Due to the roles of *MYC* and *TERT* in lung cancer, the aims of this study were the verification of our recent results analyzing *MYC* CNV in tumor and non-tumor tissue of lung cancer patients using an independent study group and the assessment of *TERT* CNV as an additional marker.

## Main text

### Methods

#### Study group

Between 08/2017 and 06/2020, 423 subjects were recruited in the Malteser Krankenhaus Seliger Gerhard Bonn/Rhein-Sieg. Of those, within the context of surgical interventions, lung tissue samples were collected from 144 lung cancer patients, immediately cooled to 4 °C and washed with isotonic saline solution. Pathological evaluation was performed and finally, 114 primary tumor and adjacent non-tumor tissues without signs of inflammation were selected as appropriate for CNV analyses. Samples were stored at − 80 °C until analyses.

All participants of the study provided informed consent. The study was designed according to the rules guarding patient privacy and with the approval from the ethics committee of the Faculty of Medicine, Ruhr University Bochum (registration number 4552-12) and performed in accordance with the Declaration of Helsinki.

#### Detection of CNV

Isolation of genomic DNA (gDNA) was performed from two 40 µm sections of frozen tissue using the QIAamp Fast DNA Tissue Kit (Qiagen, Hilden, Germany) as recently described [7]. Notably, isolated gDNA was not pretreated, i.e., fragmented, according to Brik et al. [7]. Digital PCR (dPCR) was carried out using the QuantStudio 3D Digital PCR 20 K Chip Kits v2 (AppliedBiosystems, Pleasanton, CA, USA) with 35 ng gDNA as template according to the manufacturer’s instructions using FAM-labeled *TERT* (Hs06005815_cn; Thermo Fisher Scientific) and *MYC* (Hs02758348_cn; Thermo Fisher Scientific) as marker, and VIC-labeled *RNase P* (4403326; Thermo Fisher Scientific) as reference. All assays were performed using duplex assays, i.e., *RNase P* and *MYC* and *TERT*, respectively, were determined in parallel within a single reaction. CNV was calculated as 2 × ratio of the *MYC* and *TERT* copy number, respectively, to the *RNase P* copy number.

#### Statistical analyses

Statistical analyses were performed using SAS software, version 9.4 (SAS Institute Inc., Cary, NC, USA). Dot plots with median and inter-quartile range (IQR) were used to depict the distribution of single markers. Mann–Whitney U tests were applied to examine group differences and Wilcoxon signed-rank tests to compare two related samples. P < 0.05 were considered as statistically significant. Receiver operating characteristic (ROC) curves were used to quantify classification performance and cut-offs of single markers. The accuracy of the diagnostic tests was depicted by the area under curve (AUC) and its 95% confidence interval (CI). Marker cut-offs were determined by the highest sensitivity at fixed specificities of 99%. The chi-squared test was used to compare assays status and clinicopathological parameters.

Combinations of *MYC* and *TERT* were evaluated using the two Boolean operators AND and OR to combine both markers. Applying the AND operator, the two-marker combination was defined as positive if both markers were positive, whereas applying the OR operator the two-marker combination was defined as positive if at least one of the two markers was positive. For both operators, the cut-off values correspond to the highest sensitivity at 99% specificity of all potential cut-off combinations.

### Results

#### Study population

In Table 1 the clinicopathologic parameters of the included 114 lung cancer patients are presented. Median age of the patients was 70 years. Most of the participants were current (47.4%) or former smokers (35.9%), whereas 14.9% were never smokers. Mostly, lung cancer patients were diagnosed with LUAD (64.9%) or LSCC (23.7%) and the majority of patients (71.9%) were diagnosed with tumor stage T1 or T2.

**Table 1:** Patients´ characteristics and clinicopathological parameters in the study group of 114 lung cancer patients

Characteristic	Group	N (%)
Age (years), median (range)		70 (50–85)
Sex	Male	57 (50.0)
	Female	57 (50.0)
Smoking status	Never	17 (14.9)
	Former	41 (35.9)
	Current	54 (47.4)
	Missing	2 (1.8)
Histological subtypes	Lung adenocarcinoma	74 (64.9)
	Lung squamous cell carcinoma	27 (23.7)
	Other^#^	13 (11.4)
Grade^§^	G1	5 (4.4)
	G2	50 (43.9)
	G3	52 (45.6)
	GX	7 (6.1)
T Stage	T1	43 (37.7)
	T2	39 (34.2)
	T3	16 (14.0)
	T4	11 (9.7)
	TX	5 (4.4)

^#^four carcinoid, three large cell neuroendocrine carcinomas, two non-small cell lung cancer-not otherwise specified, two adenosquamous carcinoma, one large cell carcinoma-not otherwise specified and one carcinosarcoma of the lung

^§^Grade 1: well differentiated, low grade; Grade 2: moderately differentiated, intermediate grade; Grade 3: poorly differentiated, high grade; Grade X: grade cannot be assessed (undetermined grade)

#### Assessing of *TERT* and *MYC* copy number

**Fig. 1:**
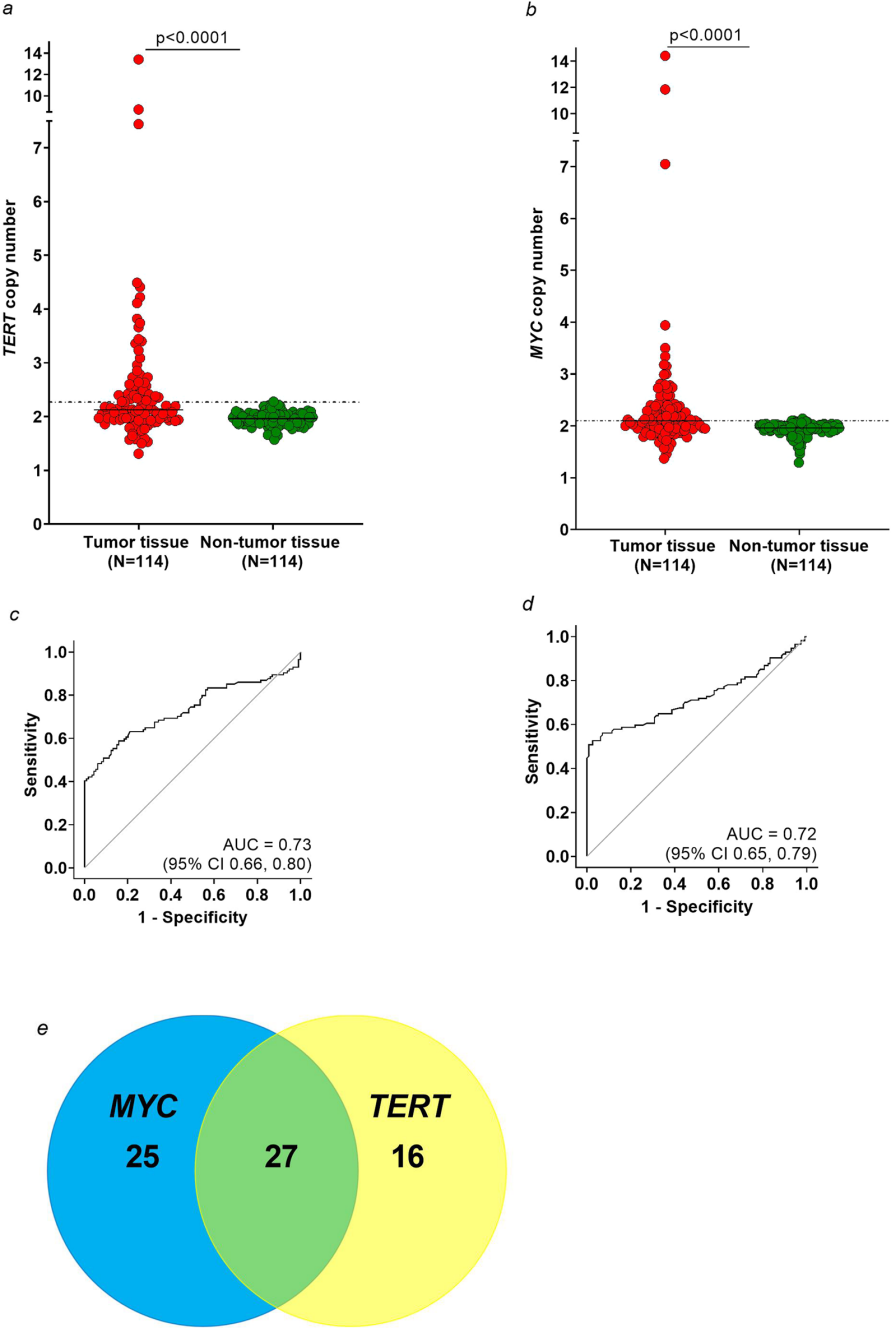
**a**–**b**: Distribution of copy number variation (CNV) of *TERT* (**a**) and *MYC* (**b**) in tumor (red) and non-tumor (green) tissue samples from 114 lung cancer patients. The dotted lines indicate the cut-offs for *TERT* (2.27) and *MYC* (2.10) and the horizontal lines the median. **c**–**d**: Receiver operating characteristic (ROC) analysis for *TERT* (**c**) and *MYC* (**d**) based on tumor and adjacent non-tumor tissues from the 114 lung cancer patients. **e**: Venn diagram of the number of true positive tests in lung cancer patients using *MYC* (blue) and *TERT* (yellow)

The median *TERT* copy number in tumor samples was 2.12 (IQR 1.96–2.56) and in non-tumor samples 1.96 (IQR 1.88–2.04). The difference between tumor and non-tumor samples was statistically significant (p < 0.0001), (Fig. 1a). The highest *TERT* copy number was 13.40 in tumor tissue and 2.28 in non-tumor tissue. Regarding the histological subtype, differences between LUAD and LSCC samples and their corresponding non-tumor samples were also statistically significant (p < 0.0001 and p = 0.0002, respectively), (Additional file 1: Figure S1a–b).

For *MYC* the median copy number was 2.10 (IQR 1.94–2.38) in tumor tissues and 1.96 (IQR 1.89–2.03) in non-tumor tissues and the difference between tumor and non-tumor was statistically significant (p < 0.0001), (Fig. 1b). The highest *MYC* copy number in tumor samples was 14.41 in contrast to 2.14 in non-tumor samples. Differences between LUAD and LSCC tissues and their corresponding non-tumor tissues were statistically significant (p < 0.0001 and p = 0.0010, respectively), (Additional file 1: Figure S1c–d).

Differences of copy numbers between the two histological subtypes LUAD and LSCC were not statistically significant, neither for *MYC* nor for *TERT*.

For the differentiation between tumor and non-tumor tissue the ROC analysis revealed an AUC of 0.73 (95% CI 0.66, 0.80) for *TERT* (Fig. 1c) and 0.72 (95% CI 0.65, 0.79) for *MYC* (Fig. 1d). Using a predefined specificity of 99% (cut-off 2.27) a sensitivity of 41% was observed for *TERT*. Accordingly, *TERT* was amplified in 47 of 114 tumor tissues of lung cancer patients. Regarding the histological subtype, *TERT* was amplified in 30 of 74 LUAD (40.5%) and 13 of 27 LSCC cases (48.1%), (Table 2).

**Table 2:** Distribution of clinicopathological characteristics regarding different CNV cut-off values in tumor tissue

Characteristic	Group	N	*MYC* ≥ 2.10^A^ (N = 58, 50.9%)	*MYC* < 2.10^A^ (N = 56, 49.1%)	p-value (chi^2^ test)	*TERT* ≥ 2.27^B^ (N = 47, 41.2%)	*TERT* < 2.27^B^ (N = 67, 58.8%)	p-value (chi^2^ test)
Age	≤ 70 years	62	31 (53.5)	31 (55.4)	0.8379	24 (51.1)	38 (56.7)	0.5509
	> 70 years	52	27 (46.6)	25 (44.6)		23 (48.9)	29 (43.3)	
Sex	Female	57	22 (37.9)	35 (62.5)	0.0087	21 (44.7)	36 (53.7)	0.3414
	Male	57	36 (62.1)	21 (37.5)		26 (55.3)	31 (46.3)	
Smoking status	Never	17	6 (10.3)	11 (19.6)	0.3230*	6 (12.8)	11 (16.4)	0.0590*
	Former	41	21 (36.2)	20 (35.7)		12 (25.5)	29 (43.9)	
	Current	54	29 (50.0)	25 (44.6)		27 (57.5)	27 (40.3)	
	Missing	2	2 (3.5)	0 (0.0)		2 (4.3)	0 (0.0)	
Histological subtypes	Lung adenocarcinoma	74	36 (62.1)	38 (67.9)	0.2827	30 (63.8)	44 (65.7)	0.5670
	Lung squamous cell carcinoma	27	17 (29.3)	10 (17.9)		13 (27.7)	14 (20.9)	
	Other	13	5 (8.6)	8 (14.3)		4 (8.5)	9 (13.4)	
Grade	G1 + G2	55	24 (41.4)	31 (55.4)	0.0399	23 (48.9)	32 (47.8)	0.6515
	G3	52	33 (56.9)	19 (33.9)		24 (51.1)	28 (41.8)	
	GX^§^	7	1 (1.7)	6 (10.7)		0 (0.0)	7 (10.5)	
T Stage	T1	43	22 (37.9)	21 (37.5)	0.6470	15 (31.9)	28 (41.8)	0.3371
	T2	39	23 (39.7)	16 (28.6)		18 (38.3)	21 (31.3)	
	T3 + T4	27	13 (22.4)	14 (25.0)		14 (29.8)	13 (19.4)	
	TX^$^	5	0 (0.0)	5 (8.9)		0 (0.0)	5 (7.5)	

^A^cut-off value for *MYC* at 99% specificity and 51% sensitivity

^B^cut-off value for *TERT* at 99% specificity and 41% sensitivity

^§^GX was excluded from tests

^$^TX was excluded from tests

^*^p-value from Fisher’s exact test

For *MYC* 51% sensitivity was observed using the predefined specificity of 99% (cut-off 2.10). Thus, *MYC* was amplified in 58 of 114 tumor tissues of lung cancer patients. Regarding the histological subtype *MYC* was amplified in 36 of 74 LUAD (48.6%) and in 17 of 27 LSCC cases (63.0%), (Table 2).

For the combination of *TERT* (cut-off 2.29) and *MYC* (cut-off 2.14) the overall sensitivity increased to 60% at the predefined specificity of 99% using the OR operator, whereas a sensitivity of 39% at 99% specificity was revealed using the AND operator. Thus, using the OR combination for *TERT* and *MYC*, a total of 68 lung cancer tissues were identified correctly, 16 samples by *TERT,* 25 by *MYC*, and 27 by both markers (Fig. 1e). In particular, the OR combination detected 44 of 74 LUAD (59.5%) and 18 of 27 LSCC cases (66.7%) correctly.

#### Influence of clinicopathological parameters on *TERT* and *MYC*

No significant group difference between tumor tissue and non-tumor tissue with regard to age, smoking status, tumor histological subtype, grading, and T stage could be observed for *TERT,* whereas *MYC* showed statistically significant differences between males and females (p = 0.0217) and regarding grading, i.e., G1 + G2 vs. G3 (p = 0.0399) (Table 2).

## Discussion

Amplification of *MYC* and *TERT* is a common event in lung cancer. Thus, the assessment of *TERT* and *MYC* CNV might be meaningful for the application in clinical diagnostics. In this study, we observed a *TERT* amplification in 41% of the lung cancer samples, confirming recent studies showing an amplification of *TERT* in lung cancer cases between 38% [18] and 57% [9]. In contrast to *TERT* the published percentages for amplification of *MYC* in lung cancer vary more, ranging from 11% [19] to 88% [20]. In this study, *MYC* was amplified in 51% of the lung cancer cases, verifying our former results (43% sensitivity and 99% specificity) in an independent study group [7]. Both study groups are comparable regarding most of the clinicopathological parameters. Differences between our two study groups exist only regarding sex, but this difference may be due to the relatively small number of samples in both study groups. However, Li et al. have showed that amplification of *MYC* is more frequent in males (48%) than in females (37%) [21]. Thus, a detailed analysis regarding CNV and sex should be performed in the future.

Generally, the combination of markers within a panel has the potential to increase the performance of the single markers. For example, in lung tumors a panel of the four markers *MYC*, *TP63*, *CEP3*, and *CEP6* showed an improved performance in comparison to each individual marker [22]. In accordance, in this study the combination of *MYC* and *TERT* lead to an increase of sensitivity to 60% to detect lung cancer, suggesting that the combination of both markers could be useful for the differentiation between lung cancer and normal tissue.

Regarding the histological subtypes, *TERT* amplification was detected in 41% of the LUAD and 48% of the LSCC cases. Barthel et al. observed *TERT* amplification in 13% of LUAD and 14% of LSCC cases [12], whereas other studies detected a *TERT* amplification in 83.8% [23] and 74.8% [24] of the analysed LUAD patients [23, 24]. Similarly, *MYC* is amplificated in 45% of the LUAD and 59% of the LSCC cases in this study. This is in accordance with Han et al. observing a *MYC* amplification in 68.1% of LUAD [24].

Despite the general discrimination between lung cancer and normal tissue *MYC* and *TERT* CNV are appropriate to distinguish between each histological subtype and normal tissue, but not to discriminate LUAD from LSCC.

Besides their use as diagnostic markers, the assessment of *MYC* and *TERT* in tissue might also be meaningful for the application as prognostic markers in lung tumor patients. *MYC* is a promising marker candidate for LUAD and associated with poor prognosis [25] and Flacco et al. have shown that an increase of *MYC* copy number is a predictor of worse survival in NSCLC [26]. Amplification of *TERT* may be a marker for poorer prognosis in early-stage NSCLC [9] and Liu et al. demonstrated prognostic significance for *TERT* CNV, showing that *TERT* was associated with a 35% risk reduction of LUAD progression [23]. Additionally, for ovarian cancer it was shown that *TERT* and *MYC*, as well as *PIC3CA*, *CCNE1*, and *KRAS*, might be useful to tailor therapeutics in precision medicine [27], and the same might be true for lung cancer. Alidousty et al. have shown that patients with amplification of both *MYC* [28] and *TERT* [29] are prone to fast development of resistance to ALK inhibitors after treatment of lung tumor. This should facilitate further assessment of *TERT* and *MYC* for prognostic or predictive purposes.

We are currently performing a follow-up of our patient group in order to update information on progression, recurrence, and survival. This should facilitate further assessment of *TERT* and *MYC* for prognostic purposes.

Diagnostic and prognostic use of CNV analysis of amplified genes would greatly benefit from the development of blood-based tests. A high dilution of circulating tumor DNA by normal DNA, however, still poses a challenge. The development of appropriate methods to selectively enrich tumor DNA may be a possible approach.

It was already shown, that dPCR is a valuable tool for CNV analysis [7, 30, 31] and the used method in this study confirms that the application of dPCR enables high accuracy in the detection of CNV in tumor tissues without high levels of false-positive test results in normal tissues. Thus, dPCR may be an appropriate tool in precision medicine [32–34].

## Conclusion

We demonstrated that amplification of *MYC* and *TERT* is a common event in lung cancer patients. The combination of *TERT* and *MYC* leads to an increased performance in comparison to the individual markers. For the detection of CNV, dPCR appears to be a reliable method. In the future, the assessment of *MYC* and *TERT* CNV using dPCR might be a reliable tool in precision medicine.

### Limitations

The analyzed study group is relatively small and differences of *MYC* and *TERT* detection rates in comparison to other studies are based on different group sizes, ranging from 21 [18] to 2,032 lung cancer cases [35], and different methods for the detection of CNV, dPCR [7, 32], quantitative PCR (qPCR) [9, 19], and whole genome sequencing (WGS) [2, 23, 36]. Additionally, discrepancies of *RNase P* values during determination of *MYC* and *TERT* in identical samples makes it necessary to develop appropriate verification methods. However, measurement of references in parallel to the marker using duplex assays already compensate differences during dPCR in comparison to simplex assays. However, consistency between simplex and duplex assays should be assessed in detail. Thus, larger studies with consistent methods for the assessment of CNV are needed.

Additionally, no follow-up data of the patients are available at the moment. Thus, the feasibility of *MYC* and *TERT* as prognostic markers could not be evaluated yet.

### Supplementary Information

**Additional file 1: Figure S1.** Distribution of copy number variation (CNV) of *TERT* in LUAD, *TERT* in LSCC, *MYC* in LUAD and *MYC* in LSCC in tumor and non-tumor tissue samples from 74 LUAD and 27 LSCC patients, respectively.

**Additional file 2: Table S1.** Raw Data: Levels of *RNaseP*, *MYC* and *TERT* (copies/µL) by dPCR.

## Data Availability

The raw data supporting the conclusions of this article are included as Additional file 2.

## Abbreviations

AUC: Area under the curve
Cl: Confidence interval
CNV: Copy number variations
dPCR: Digital PCR
IQR: Inter-quartile range
LSCC: Lung squamous cell carcinoma
LUAD: Lung adenocarcinomas
*MYC*: V-myc avian myelocytomatosis viral oncogene homolog
NSCLC: Non-small cell lung cancer
qPCR: Quantitative PCR
ROC: Receiver operating characteristic
*TERT*: Telomerase reverse transcriptase
WGS: Whole genome sequencing
