# Supplementary material for: Assessment of MYC and TERT copy number variations in lung cancer using digital PCR
Source: BMC Res Notes. 2023 Oct 19;16:279. doi: 10.1186/s13104-023-06566-x (PMC10585721; doi:10.1186/s13104-023-06566-x)
Supplement: Supplementary file 1 — Additional file 1: Figure S1. Distribution of copy number variation (CNV) of TERT in LUAD, TERT in LSCC, MYC in LUAD and MYC in LSCC in tumor and non-tumor tissue samples from 74 LUAD and 27 LSCC patients, respectively. [file 13104_2023_6566_MOESM1_ESM.pdf]

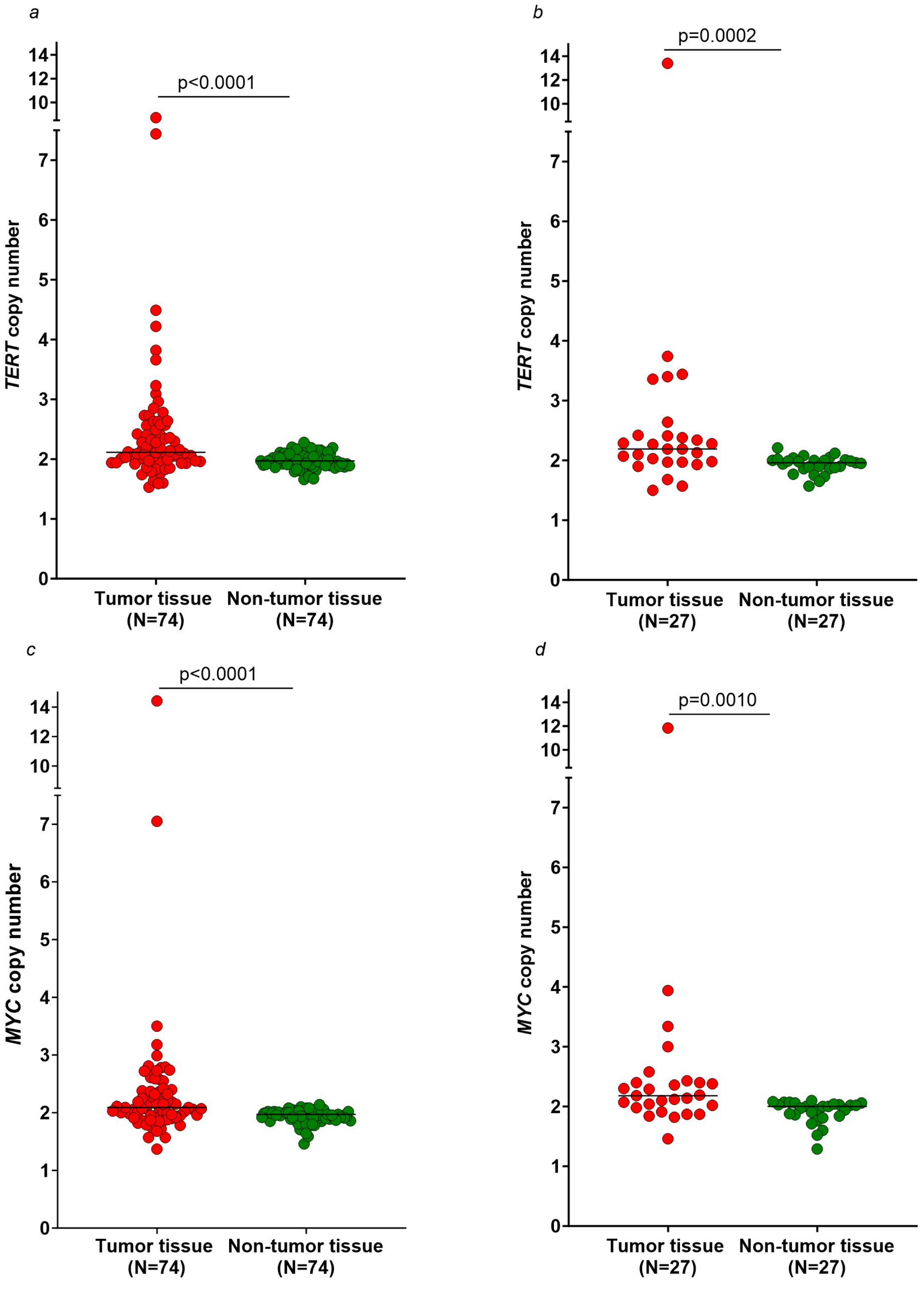

Figure S1. Distribution of copy number variation (CNV) of *TERT* in LUAD (a), *TERT* in LSCC (b), *MYC* in LUAD (c) and *MYC* in LSCC (d) in tumor (red) and non-tumor (green) tissue samples from 74 LUAD and 27 LSCC patients, respectively. Horizontal lines indicate the median.
